# Quantum Dots—From Synthesis to Applications in Biomedicine and Life Sciences

**DOI:** 10.3390/ijms11010154

**Published:** 2010-01-12

**Authors:** Gregor P.C. Drummen

**Affiliations:** 1 Bio&Nano-Solutions, Helmutstr. 3A, D-40472, Düsseldorf, Germany; E-Mail: gpcdrummen@bionano-solutions.de; Tel.: +49-211-22-973-648; Fax: +49-322-22-407-500; 2 DNA Damage and Ageing Group, University of Duisburg-Essen Medical School, Hufelandstr. 55, D-45122, Essen, Germany

**Keywords:** nanotechnology, nanomedicine, bionanotechnology, nanoparticle, quantum dot, fluorescence, flow cytometry, toxicity, imaging

## Abstract

Imagine devices or particles so small that they are invisible to the naked eye. Imagine that such entities could be used to patrol our bodies and autonomously augment endogenous defense and repair mechanisms. Imagine the defeat of illness at a fraction of the current costs. Bionanotechnology is the field of science that deals with just that: the development of imaging, tracking, targeting, sensing, diagnostic, and eventually therapeutic capabilities based on particles in the nanometer range, *i.e.*, “nanoparticles”. Within the extensive group of nanoparticles, semiconducting quantum dots play a central and prominent role. Quantum dots excel at a myriad of physical properties, most notably their fluorescent properties, such as high quantum yield, photo-stability, broad absorption spectra, and their remarkable size-dependent emission-tunability.

## 1. Introduction

From its first conception, envisioning technology beyond the microscopic level, below approximately 100 nm, nanotechnology has seen slow but steady development over the past five decades. Nanotechnological concepts and the fascinating possibilities that might arise when it would become possible to manipulate matter at the atomic scale were originally described in a lecture titled “There’s plenty of room at the bottom”, delivered at an American Physical Society meeting in 1959 by the physicist Richard Feynman, a Nobel Prize-laureate in 1965. The term “nanotechnology” was first defined by Norio Taniguchi in 1974 when he wrote that the term was derived from nanometer and defined “nanotechnology” as follows: “Nanotechnology mainly consists of the processing of, separation, consolidation, and deformation of materials by one atom or by one molecule” [1]. The nanoscale—one billionth of a meter (10^−9^ m)—marks the hazy boundary between the classical, Newtonian, and quantum mechanical worlds, and thus a variety of unique and deviant physical and chemical properties arise in nanoparticles compared with bulk materials.

**Figure 1.**
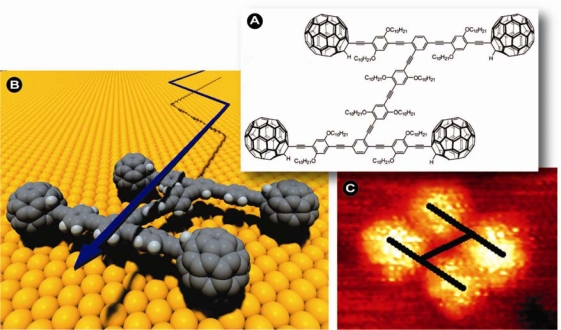
An example of nanotechnological progress, *i.e.*, a thermally driven single-molecule nanocar capable of moving on a gold surface. (a) Structure of the nanocar (nanocar-1); fullerene wheels and chassis parts can rotate because of the alkyne connections, giving the nanocar the ability to roll on the surface and flexibility orthogonal to the surface plane. Synthesis and study of several related structures clarified that the alkyl units were critical for the solubility of the molecule. (b) Three-dimensional model and summary of the method of motion for nanocar-1, which consecutively pivots and then translates perpendicular to its axes. For clarity, the structure is drawn devoid of the alkoxy units. (c) High resolution scanning tunneling microscope (STM) image of nanocar-1 on an Au(111) surface (*V*_b_ = 0.4 V, *I*_t_ = 60 pA). Bright features are fullerene wheels; intramolecular oligo(phenylene ethynylene) and alkyl groups are not visible. Images courtesy of James M. Tour and reprinted with permission from [2] (© 2005 American Chemical Society).

At the outset it was envisioned that nanoscopically small devices could replace conventional industrial production methods and since such machines may convert matter at the atomic level to the desired end-product, a high substrate and product specificity, energy and conversion efficiency, and thus very little to no waste might be expected. These goals are still being pursued with significant scientific vigilance, but it is in a sense rather fitting that developments have been fastest in the biological and biomedical field, where substrate specificity, conversion efficiency, and product stereo-selectivity play crucial roles and are unparalleled endogenous biological traits. That such small devices are by no means the vision of mad scientists or science fiction writers, but already a reality is for instance exemplified by the engineering of nano-cars (Figure 1): molecular chassis with fullerene wheels capable of motion and even directional control [2].

In the biomedical field, nano-devices that might augment natural healing or defense processes, that could perform microsurgical tasks, actively and intelligently eradicate aberrant cells and tissues would constitute a revolution that surpasses all the milestones of the last centuries, such as the discovery in 1840 that ether gas can be used as a general anesthetic by Crawford Long and William Morton, Louis Pasteur’s unveiling of the artificial generation of weakened microorganisms in 1885—the principle was first established for the rabies vaccine—that paved the way for every other vaccine, or even the discovery of the first antibiotic, penicillin, by Sir Alexander Fleming in 1928. All these feats have saved the lives of millions of people.

Small nanoscopic medical devices that intelligently watch over the health of the individual—the stuff that Sci-Fi dreams are made of, such as the 1966 movie Fantastic Voyage, where Richard Fleischer envisioned to send surgeons in their miniaturized submarine into the body of an escaped Czech scientist to remove a blood clot in his brain, or more aptly considering the scope of this editorial, the “nanites” that autonomously regenerate tissue in injured Borg, used in Gene Roddenberry’s Star Trek series—are perhaps a long way of, but it is necessary to envision such goals for progress’ sake. On the other hand, the first “small step” may already have preceded “the giant leap” in current developments in nanoparticles that combine detectability, targeting, and therapeutic options in a single particle, *i.e.*, “multifunctional” or “multimodal” probes. I personally prefer the latter term. Whether it is justified to already call this “thera(g)nostics” or not is a matter of debate, but fact is that such attributes have been demonstrated in a variety of nanoparticles by a number of laboratories.

**Figure 2.**
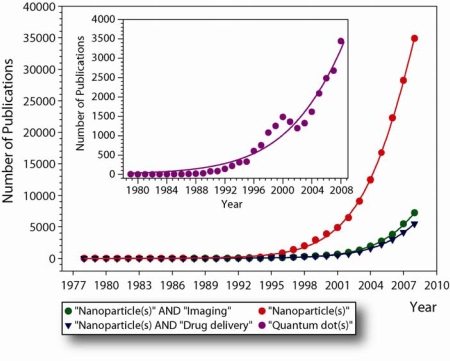
Overview of the number of publications on nanoparticles until 2009 from SCOPUS^™^ as a measure for the development of the bionanotechnological field over the past three decades [3]. Notice that currently the number of publications released show exponential growth dynamics. Similar trends were obtained when using NCBI/Pubmed queries.

Developments in this novel field of science at the boundary between physical, chemical, biological and medical science, *i.e.*, “Bionanotechnology”, have been relatively slow at the beginning, but momentum is taken up and current developments are progressing exponentially, as semi-empirically deducible from the increase in the yearly number of publications (Figure 2). This normally is an indication that a new technology is maturing and this is certainly the case for a number of nanoparticles currently employed for research purposes. Evolutionary progress is complementary in the fields of nanotechnology and biotechnology, and there is a constant technological exchange and traffic between these. Nanoparticles are not only used to elucidate and study biological processes, but biomolecules, viruses, and cells are being used to fabricate and organize nano-materials that could not be produced *via* synthetic techniques alone. The impact that nanostructured materials are already having on biological and biomedical research is profound. One of the most exciting applications are imageable nanoparticles, foremost fluorescent semiconducting quantum dots (Qdots).

## 2. Semiconductor Quantum Dots

**Figure 3.**
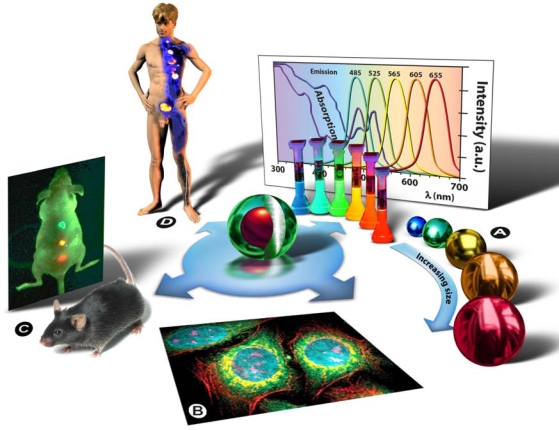
Quantum dots as multifunctional research and diagnostic tools. Centre: schematic representation of a core/shell quantum dot. (a) Illustration of how the physical size of the quantum dot determines its fluorescent properties: the larger the Qdot, the larger the Stokes-shift, *i.e.*, the redder the emission. Also note from the broad absorption spectra in the back that virtually all Qdots can be excited simultaneously with one wavelength in the violet to UV region of the electromagnetic spectrum. (b) Multiplex pseudocoloured image of cellular structures visualized with quantum dots. Fixed human epithelial cells were stained to show the nucleus (blue; 655-nm Qdots), Ki-67 cell proliferation proteins in the nucleus (magenta; 605-Qdots), mitochondria (orange; 525-Qdots), microtubules (green; 565-Qdots), and actin filaments (red; 705-Qdots). Courtesy of Quantum Dot Corp./Invitrogen. (c) Example of Qdot-based imaging capabilities in intact animals by simultaneous imaging of multicolor Qdot-encoded microbeads (0.5 μm diameter) emitting green, yellow or red light. Approximately 1–2 million beads in each color were injected subcutaneously at three adjacent locations on a host animal. Notice the significant brightness and spectral shifting advantages away from auto-fluorescence. Adapted with permission from Macmillan Publishers Ltd: Nature Biotechnology [16], (© 2004). (d) The ultimate goal would be to use functionalized Qdots for the simultaneous detection and concomitant treatment of disease states in human patients.

Semiconducting nanocrystal technology was initially developed in the early 1980s in the labs of Louis Brus at Bell Laboratories [4] and of Alexander Efros and Alexei I. Ekimov [5] of the A.F. Ioffe Physical-Technical Institute in St. Petersburg. The term “quantum dot” was first contrived by Mark A. Reed in 1988 and denotes nanocrystalline semiconducting fluorophores, whose excitons are confined in all three spatial dimensions—quantum confinement: strict confinement of electrons and holes when the nanoparticle radius is below the exciton Bohr radius—and have typical diameters of 2–20 nm. Generally they are binary systems composed of a core of semiconducting material enclosed within a shell of another semiconductor (Figure 3: Centre). Qdot fluorescence is caused by the bandgap between the valence and the conduction electron bands, and absorption of a photon higher in energy than the spectral bandgap of the core semiconductor results in electron excitation to the conduction band, generating an electron-hole pair (exciton). The long lifetime in the order of 10–40 ns increases the probability of absorption at shorter wavelengths, and produces a broad absorption spectrum (Figure 3A). Since the physical size of the bandgap determines the photon’s emission wavelength, it is possible to control the fluorescence wavelength by the size of the nanoparticle (the bandgap energy is inversely proportional to the square of the size of the quantum dot). Simply put: the larger the Qdot, the redder its emission (Figure 3A). Qdots are characterized by a number of unique physical properties, but since they are currently predominantly used as imaging agents it is particularly worth mentioning their optical properties: strong light absorbance, size-tunable emission, bright fluorescence/high quantum yield, narrow symmetric emission bands, high photostability and low photobleaching rates, and their broad absorption spectrum allows the simultaneous excitation of Qdots of all sizes by a single excitation light source in the UV to violet part of the spectrum (Figure 3A).

Chemical adaptation of the nanoparticle surface not only renders the particle water-soluble, allows biocompatiblilization and functionalization, but also eliminates photobleaching by physically excluding interaction of the excited state particle with molecular oxygen and thus prevents formation of reactive oxygen species, such as singlet oxygen. By attaching particular targeting molecules, Qdots can be directed to sub-cellular structures (Figure 3B) or even aberrant tissues in intact animals (Figure 3C), and therefore can be used for bio-imaging purposes in cells [6] and animals [7,8]. Next to bio-imaging applications, Qdots are currently being used in bio-analytical assays [9] with a pure research focus. Ultimately, such strategies might be used in human patients to localize and treat diseased tissues and organs, as depicted in figure 3D, or to replace conventional methods in clinical assays for diagnostic purposes. Drug delivery and therapeutic applications, such as photodynamic therapy [10] and gene silencing [11,12], as well as applications that combine imaging capabilities with delivery of a therapeutic payload are presently being developed and tested in lab-animals. Clinical applications and trails in humans have not yet been performed pending resolution of remaining issues, such as tissue targeting, renal clearance, and toxicity issues.

## 3. Quo Vadis Quantum Dots

This special edition of the *International Journal of Molecular Sciences* (*IJMS*) brings together a number of articles dedicated to quantum dots and their application in life and biomedical sciences. Collectively, these articles illustrate recent advances in the field and highlight a promising and bright future for life- and biomedical sciences as well as the nanotechnological sciences that created them in the first place.

Maureen Walling and Jennifer Novak from Jason Shepard’s group, University at Albany (US) extensively review the application of quantum dots in live cell and *in vivo* imaging [13]. Their review focuses on imaging applications in single cell microscopy, tracking of individual cells, e.g., metastasis, and whole animal imaging and concomitant problems, as well as tackling the issue of targeting Qdots to particular (sub)cellular structures, cells, and tissues.

Jana Drbohlavova, Vojtech Adam, Rene Kizek, and Jaromir Hubalek, Brno University of Technology (CZ), center their review on the preparation and characterization of Qdots [14]. The authors formulate several criteria for the safe use of Qdots in medicine and discuss short-comings in the current generation of Qdots.

Gerard Giraud *et al.* from the University of Edinburgh (UK) in a collaborative effort between the Institute for Condensed Matter and Complex Systems, the Division of Pathway Medicine, the School of Chemistry, the Institute of Integrated Systems, and the National Physical Laboratory, describe the use of Qdot labeling combined with fluorescence lifetime imaging microscopy for the detection of DNA hybridization events on DNA microarrays [15]. The authors show in this paper that exploitation of the relatively long lifetime of quantum dots with a FLIM imager that combines evanescent wave excitation with wide field detection and a quadrant anode mounted on an inverted microscope can be used to increase the contrast ratio of DNA microarrays by a factor of two.

In their paper, Raquel Ibáñez-Peral *et al.* from the departments of Chemistry and Biomolecular Sciences, Earth Sciences, and Physics from Macquarie University and the School of Biotechnology and Biomolecular Sciences, University of New South Wales in Sydney (AU) investigate whether Qdots can be used as suitable multicolor optical labels of specific nucleotide probes for microbial identification using Flow Cytometry (FCM) [17]. Since individual Qdots fluoresce and scatter below the resolution of the flow cytometer, the authors conjugated Qdots to paramagnetic beads (Dynabeads). They conclude that the minimum fluorophore-concentration required for detection of Qdots above the autofluorescent background was 100-fold less than for the commonly used fluorophore FITC, even under suboptimal excitation conditions. Nonetheless, their research also showed that Qdot binding to the beads markedly influences their optical properties and this fact alone suggests that the application of Qdots for FCM needs further study and development.

Takashi Jin and Yoshichika Yoshioka *et al.* from the WPI Immunology Frontier Research Center, Osaka University (JP), in an excellent paper, describe preparation and characterization of glutathione (GSH)-coated near-infrared (NIR) Qdots for *in vivo* fluorescence imaging [18]. NIR probes are increasingly being used for deep tissue imaging, because they emit outside the autofluorescence window, and excitation beams in this region of the electromagnetic spectrum display better tissue penetrability and reduced photon scattering. Jin *et al.* developed an easy synthesis route for GSH-coated NIR Qdots with a core-shell structure (CdSeTe/CdS) that can be used in cellular imaging, but also in intact animals, as demonstrated by lymph node imaging in a C57BL/6J mouse—the most widely used inbred strain refractory to many tumors, characterized by resistance to audiogenic seizures, relatively low bone density, development of age-related hearing loss, susceptibility to diet-induced obesity, type 2 diabetes, and atherosclerosis [19].

To conclude, Ming-Shu Hsieh and Nion-Heng Shiao from Wen-Hsiung Chan’ lab, Department of Bioscience Technology and Center for Nanotechnology, Chung Yuan Christian University (TW), report on the influence of CdSe-core Qdots on mouse oocyte maturation, fertilization, and subsequent pre- and post-implantation fetal development [20]. The authors show that exposure of mouse oocytes to CdSe-core Qdots during *in vitro* maturation decreases cell number, induces apoptosis, and inhibits post-implantation development. Encapsulation with a ZnS shell effectively abrogates cytotoxic and teratogenic effects, demonstrating not only the potential toxicity in type I Qdots, but also the influence of the outer shielding.

## 4. Concluding Remarks

Bionanotechnology has undeniably arrived, but it has yet to realize its full potential. Since developments in this adolescent field of science are irrefutably moving at exponential rates (Figure 2), the next decade will be extremely exciting for those of us working at the interface of nanoscience, chemistry, medicine, and biology. Similar quantum leaps may be anticipated as those made in the field of molecular biology and biotechnology around the turn of the millennium and perhaps one day small medical devices in our circulation will augment natural biological defense and repair systems as envisioned by science fiction.

Currently, we are at a stage where a myriad of nanoparticles have been developed: Qdots that give away the location of cancer cells in tissues of higher organisms, nanoparticles that deliver therapeutic drugs directly to target cells for *in situ* delivery and as such minimize damage to healthy cells, nanoshells that concentrate the heat from infrared light to destroy cancer cells with minimal damage to surrounding healthy tissue (thermal ablation), and others. The series of papers in this special edition highlight the rapid progress in this field and it is hoped that the next generation of nanoparticles will be a major asset in the fight against numerous diseases, especially cancers. However, nanoparticles currently employed can hardly be called “intelligent” and the coming generations of nanoparticles will certainly include logic decision mechanisms in order to smarten up such particles. The first steps toward such systems are already under way, as exemplified by the recent paper by Motornov *et al*., who coupled pH-responsive nanoparticles with enzyme-based “logic gates” that were able to perform AND/OR logic operations in which biochemical input signals were transduced into an output signal (pH change) sufficient to induce reversible structural changes of the nanoparticle assemblies [21]. Their proposed scheme enabled the processing and amplification of biochemical signals.

As a final point, logic dictates a note of caution: the deviant behavior of nanoparticles compared with bulk materials not only brings about extraordinary physical and chemical properties and as such provides the basis for their implementation and success in biological and biomedical research, but concomitantly threatens the safe use of such nanomaterials in a clinical setting. There are now numerous studies that indicate that nanoparticles of various forms not only potentially exhibit cyto- and genotoxic effects [22,23], threaten fertility and fetal development [20], but also physically disrupt biomembranes causing changes in fluidity, cell membrane permeability, nanoparticle adsorption onto or intercalation into the lipid bilayer, with possibly detrimental cellular effects [24,25]. Extensive and long term health and toxicity studies are rare, let alone studies that focus on environmental impact and particularly ecosystem effects. This might be a cause for concern, especially since nanoparticles are being generated in all flavors, from a myriad of raw materials, at an ever increasing speed and already supposedly have silently found their way into cosmetics and foodstuffs without such studies. Such news flashes have understandably led to considerable consternation in post-BSE Europe more so than anywhere else in the world. Perhaps those doing the engineering and science should more closely collaborate with toxicological and environmental research groups, whilst governmental agencies and institutions should be more aware of the potential dangers, in order to steer clear of errors made in the past with so many new technologies and avoid “end-of-pipe” solutions. If one thing has become clear regarding technological advances in the last century, mopping up is always a worse scenario than clever engineering from the bottom up. Nonetheless, it is my opinion that bionanotechnology and quantum dots in particular, have a bright future and that it will carry us far on the road of progress, provided we use our common sense.
